# Searching out the Secrets of Nature

**Published:** 1899-11-04

**Authors:** 


### SEARCHING OUT THE SECRETS OF NATURE.

The Hon. Stephen Coleridge writes: In your leading article of October 28th on Dr. Poore's Harveian oration you challenge anti-vivisectors to come into the Law Courts and submit themselves to cross-examination. I for one am ready to do so as soon as any vivisector elects to prosecute me for libel. I have frequently published detailed accounts culled from the writings of the physiologists describing the tortures they have inflicted upon their victims. If I have made a false quotation they have their remedy; they know where I live. To twit us with not prosecuting them for these cruelties is absurd, because everybody knows that under the present law these acts of the vivisectors become legal if their perpetrator holds a certificate issued by the Home Office.

*** The Hon. Stephen Coleridge is hardly accurate. We threw down no challenge to the anti-vivisectors, and we certainly had no wish to "twit" them. What we did was to draw attention to what we considered equivalent to a challenge on the part of Dr. Vivian Poore. We are glad, however, to have Mr. Coleridge's assurance that the acts of which the anti-vivisectors complain are strictly legal.

